# Socioeconomic differences in lack of recreational walking among older adults: the role of neighbourhood and individual factors

**DOI:** 10.1186/1479-5868-6-1

**Published:** 2009-01-05

**Authors:** Carlijn BM Kamphuis, Frank J van Lenthe, Katrina Giskes, Martijn Huisman, Johannes Brug, Johan P Mackenbach

**Affiliations:** 1 Department of Public Health, Erasmus University Medical Centre, P.O. Box 2040, 3000 CA Rotterdam, The Netherlands; 2 School of Public Health/Institute of Health and Biomedical Innovation, Queensland University of Technology, Victoria Park Road, Kelvin Grove, QLD 4059, Brisbane, Queensland, Australia; 3 Department of Psychiatry, University Medical Centre Groningen, P.O. Box 30001, 9700 BB Groningen, The Netherlands; 4 EMGO Institute, VU University Medical Centre, P.O. Box 7057, 1007 MB Amsterdam, The Netherlands

## Abstract

**Background:**

People with a low socioeconomic status (SES) are more likely to be physically inactive than their higher status counterparts, however, the mechanisms underlying this socioeconomic gradient in physical inactivity remain largely unknown. Our aims were (1) to investigate socioeconomic differences in recreational walking among older adults and (2) to examine to what extent neighbourhood perceptions and individual cognitions regarding regular physical activity can explain these differences.

**Methods:**

Data were obtained by a large-scale postal survey among a stratified sample of older adults (age 55–75 years) (N = 1994), residing in 147 neighbourhoods of Eindhoven and surrounding areas, in the Netherlands. Multilevel logistic regression analyses assessed associations between SES (i.e. education and income), perceptions of the social and physical neighbourhood environment, measures of individual cognitions derived from the Theory of Planned Behaviour (e.g. attitude, perceived behaviour control), and recreational walking for ≥10 minutes/week (no vs. yes).

**Results:**

Participants in the lowest educational group (OR 1.67 (95% CI, 1.18–2.35)) and lowest income group (OR 1.40 (95% CI, 0.98–2.01)) were more likely to report no recreational walking than their higher status counterparts. The association between SES and recreational walking attenuated when neighbourhood aesthetics was included in the model, and largely reduced when individual cognitions were added to the model (with largest effects of attitude, and intention regarding regular physical activity). The assiation between poor neighbourhood aesthetics and no recreational walking attenuated to (borderline) insignificance when individual cognitions were taken into account.

**Conclusion:**

Both neighbourhood aesthetics and individual cognitions regarding physical activity contributed to the explanation of socioeconomic differences in no recreational walking. Neighbourhood aesthetics may explain the association between SES and recreational walking largely *via *individual cognitions towards physical activity. Intervention and policy strategies to reduce socioeconomic differences in lack of recreational walking among older adults would be most effective if they intervene on both neighbourhood perceptions as well as individual cognitions.

## Background

Socioeconomic status (SES) is an important determinant of all cause mortality, mortality from coronary heart diseases and morbidity in many countries [1,2]. Several studies have shown that a higher prevalence of unhealthy behaviours among lower socioeconomic groups contribute to the explanation of socioeconomic inequalities in health [3-5]. Among those behaviours is physical activity, as people with a low SES are more likely to be physically inactive than their higher status counterparts [6,7]. To be able to change unhealthy behaviours in order to improve health among low SES groups, one should understand which determinants to focus on, or in other words, to understand *why poor people behave poorly *[8]. However, the mechanisms underlying the socioeconomic gradient in physical inactivity remain largely unknown. In the few studies that have attempted to explain socioeconomic differences in physical inactivity, physical environmental factors (e.g. poor neighbourhood aesthetics, safety issues, access to facilities [9,10]), social environmental factors (e.g. social participation [11]), and individual cognitions (e.g. self-efficacy or perceived behaviour control [9]) have been identified as potential explanatory factors.

**Figure 1:**
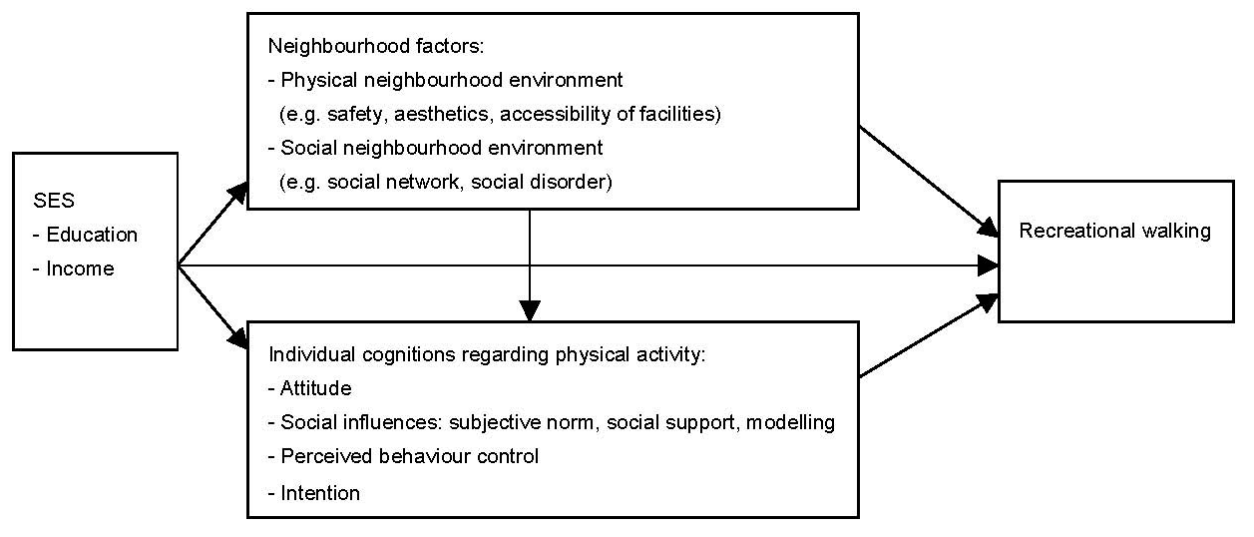
**Conceptual model of associations between socioeconomic status (SES), neighbourhood factors, individual cognitions regarding physical activity, and recreational walking**.

Few studies have simultaneously examined influences from both the environmental and individual domains, and therefore, little is known on the interplay between environmental and individual factors in the SES-inactivity relationship. As suggested in ecological models of physical activity, environmental factors may influence physical activity both directly and indirectly [12,13]. The Theory of Planned Behaviour (TPB) [14] more specifically hypothesizes how environmental factors may indirectly influence behaviours, namely via individual cognitions such as attitude, social norms and perceived behaviour control. Similarly, as shown in Figure 1, we hypothesize that environmental factors and/or individual cognitions may explain the relationship between SES and physical activity, and that environmental factors may contribute to the association between SES and physical activity directly (as stated in ecological models) or *through *individual cognitions (as stated in the TPB). For instance, people with a low SES may experience worse neighbourhood safety, and these safety concerns may reduce their perceived behavioural control expectations or have a negative impact on their attitude towards physical activity. Thus, unfavourable neighbourhood perceptions may explain the SES-inactivity relationship via low perceived behavioural control or negative attitudes, but could also have a direct effect on behaviour, e.g. when safety is perceived as barrier for doing physical activity.

Environmental determinants are likely to differ for specific physical activity behaviours, and explanatory factors to the SES-inactivity relationship may differ for population subgroups [15]. Therefore, in this paper we will focus on one specific behaviour, i.e. recreational walking, and one subgroup: older adults. Walking is the most common leisure-time physical activity among the general population in developed countries (e.g. the U.S. [16], Australia [17], and the Netherlands [18]). Walking is promising as a focus of public health interventions, due to its acceptability and accessibility (e.g. in terms of skills, equipment, and costs), especially among subpopulations who are known to be sedentary and whose activity should be increased, e.g. older people and people from a socioeconomically disadvantaged background. Older adults are an important subpopulation for public health interventions, as they represent a rapidly increasing share of the general population, and physical activity is important to preserve their health and functioning, and consequently avoid functional limitations and disability [19]. Little is known about socioeconomic differences in walking (and the determinants of these) among older adults.

In this paper we will integrate perceptions of the physical (i.e. perceived neighbourhood safety, aesthetics, and availability of facilities) and social neighbourhood environment (i.e. perceived social cohesion, social network, feeling at home in the neighbourhood, social disorganisation), with individual's cognitions regarding physical activity (e.g. attitude, perceived behavioural control), to determine to what extent socioeconomic differences in recreational walking among older adults can be explained by neighbourhood perceptions and individual cognitions.

## Methods

### Study population

Data were obtained by a large-scale postal survey, a component of the new wave of data collection for the longitudinal GLOBE study, among a stratified sample of the adult population (age 25–75 years) of Eindhoven (the fifth largest city in the Netherlands) and surrounding cities in October 2004 (N = 4785; response rate 62%). Participants resided in 213 neighbourhoods, which are the smallest geographical units in the Netherlands created for statistical and administrative purposes (with an average population of about 2000 inhabitants). More about the objectives, design and results of the GLOBE study can be found in detail elsewhere [20,21]. The use of personal data in this study is in compliance with the Dutch Personal Data Protection Act and the Municipal Database Act, and has been registered with the Dutch Data Protection Authority (number 1248943).

Participants aged 55–75 years (N = 2345) were selected for the current study. Those with missing values for recreational walking, education, household income, or sex were excluded from analyses (n = 265). Furthermore, we removed participants with missing values for the level-2 indicator (neighbourhood) (n = 26), and participants residing in neighbourhoods with only one or two participants (n = 60). Therefore, the analytic sample comprised of 1994 participants, residing in 147 neighbourhoods (mean number of participants per neighbourhood: n= 14, range 3–80). Demographic characteristics of our sample are provided in Table 1.

**Table 1:** Sample characteristics (N = 1994; aged 55–75 years) by educational level^a^, and univariate associations with no recreational walking (unadjusted).

	TOTAL		Educational level^a^				Unadjusted ORs for no recreational walking^c^
			1-low	2	3	4-high	
	N^b^	%^c^	%^c^	%^c^	%^c^	%^c^	
Total sample	1994	100					
							
Recreational walking							
Yes	1356	68.7	61.5	65.3	77.9	70.8	
No	638	31.3	38.5	34.7	22.1	29.2	
							
Education							
1 Primary education	281	12.5	-	-	-	-	1.51 (1.09–2.09)
2 Lower secondary	908	43.7					1.29 (1.01–1.64)
3 Higher secondary	366	19.8					0.69 (0.50–0.94)
4 Tertiary education	439	24.1					1.00
							
Monthly net household income							
Less than 1200 euro	294	13.8	37.4	15.6	6.2	4.5	1.33 (0.97–1.83)
1200–1800 euro	533	23.6	32.1	30.3	21.8	8.3	1.04 (0.79–1.38)
1800–2600 euro	503	25.1	11.5	26.2	32.5	24.1	0.93 (0.70–1.23)
More than 2600 euro	421	24.5	1.2	13.3	26.8	55.2	1.00
Don't want to say/don't know	243	13.0	17.7	14.7	12.7	7.9	1.57 (1.14–2.16)
							
Sex							
Male	958	47.7	42.6	32.0	59.2	69.1	1.00
Female	1036	52.3	57.4	68.0	40.8	30.9	1.06 (0.87–1.28)
							
Age group							
55–64	1053	63.5	52.5	64.4	67.4	64.2	1.00
65–74	941	36.5	47.5	35.6	32.6	35.8	0.87 (0.71–1.07)
							
Country of birth							
Netherlands	1872	93.7	87.5	97.5	95.0	89.0	1.00
Other	106	6.3	12.5	2.5	5.0	11.0	0.88 (0.59–1.32)
							
Marital status							
Married	1589	82.3	78.9	82.2	84.4	82.3	1.00
Unmarried/divorced/widowed	390	17.7	21.1	17.8	15.6	17.7	1.20 (0.94–1.53)
							
General health status							
Excellent	93	5.3	4.1	6.1	4.2	5.5	1.00
Very good	314	18.6	8.2	15.4	23.4	26.0	1.06 (0.65–1.71)
Good	1133	57.0	55.1	58.9	57.1	54.4	1.18 (0.76–1.85)
Moderate	378	15.9	26.3	16.0	13.2	12.4	1.03 (0.63–1.69)
Poor	27	0.9	2.1	0.8	0.5	0.9	0.86 (0.28–2.62)
Missing	49	2.2	4.1	2.7	1.6	0.9	2.17 (1.04–4.52)

^a^Educational level with 1 = primary education, 2 = lower secondary, 3 = higher secondary, and 4 = tertiary education.

^b ^The numbers (N) are unweighted, and reflect the actual numbers of participants in the dataset.

^c ^The percentages (%) and odds ratios (OR) are weighted and represent the prevalence rates as they existed in the population of Eindhoven by October 2004, which is the source population. The weight factors are calculated from the distribution of the characteristics in a random sample drawn from the municipal registry in Eindhoven, October 2004.

### Measures

All factors were measured in the GLOBE postal survey in 2004. Selection of items to measure salient environmental factors was based on an extensive literature review [22-25], expert meetings, and focus groups [26].

### Neighbourhood perceptions

Three perceptions of physical neighbourhood factors were measured with single items, assessing whether participants agreed or disagreed with the following statements: "My neighbourhood is unsafe" (safety), "My neighbourhood is unattractive for physical activity" (aesthetics), and "There are insufficient facilities for physical activity in my neighbourhood" (availability of facilities).

Thirteen items asked about social relationships within the neighbourhood (on a five-point scale: totally agree – totally disagree) (α = .86), and these items were represented by three factors, as derived from a factor analysis, e.g. a principal component analysis with varimax rotation and kaiser normalization. We labelled the first factor 'social cohesion', i.e. "the extent of connectedness and solidarity among groups in society" [27]. Items that loaded on this factor were e.g. 'People in this neighbourhood agree on norms and values', 'People in this neighbourhood are willing to help each other', and 'People in this neighbourhood can be trusted'. The second factor was labelled 'social network' (i.e. "the presence and nature of interpersonal relationships and interactions; extent to which one is interconnected and embedded in a community"[28]), representing items such as 'I borrow things from my neighbours', 'I visit my neighbours in their home', and 'I can ask my neighbours for advice'. The third factor was labelled 'feeling at home in one's neighbourhood', representing items such as "I feel at home in this neighbourhood", and "I would like to move out of this neighbourhood". Each factor score was classed into tertiles for analytical purposes.

The fourth social neighbourhood factor was derived from a factor analysis that grouped eleven items (α = .94) together in one factor, which we labelled as 'social disorder', i.e. "a lack of physical and social order in the community"[29]. These eleven items covered both social and physical indicators of social disorganization, and asked for the frequency with which adverse neighbourhood events occurred (often, sometimes, (almost) never). Items referred to, for instance, litter on the streets, graffiti, vandalism, and the presence of people hanging around on the streets and drinking alcohol. The factor score was classed into tertiles (high, medium, low).

### Individual physical activity cognitions

We used an adapted version of the Theory of Planned Behaviour as a framework to measure individual cognitions of regular physical activity. This expanded model incorporated the constructs of attitude, subjective norm, perceived behaviour control, and intention. Two additional social influences of physical activity were added to the model, i.e. social support, and modelling by significant others [24,28]. Items for all constructs were derived from existing scales, or formulated according to the algorithms of Conner & Norman [30]. All items were asked with regard to the behaviour "regular physical activity", which was defined in the questionnaire as "being physically active for at least 30 minutes, every day, e.g. cycling, doing sports, gardening".

Attitude was measured with outcome expectancies of regular physical activity, and responses were measured on a 5-point Likert-scale from (1) "very important" to (5) "not important at all". Participants reported on six items regarding negative outcome expectations (e.g. "Regular physical activity cost too much time", "Regular physical activity costs too much energy") and six items for positive outcome expectations (e.g. "Regular physical activity reduces my stress levels", "Regular physical activity is good for my fitness") (α = .77). Items were summed and, based on their specific sum scores, participants were divided in three groups: (very) positive attitude, positive-neutral, and neutral-negative attitude.

Social influences for regular physical activity were assessed with three separate items (α = .85) on a three-point scale (true, not true/not false, false): "Most important others (e.g. partner, children, parents, friends) think that I should be regularly active" (subjective norm), "Most important others support me to be regularly active" (social support), and "Most important others are regularly active themselves" (modelling). Items were combined into a sum score, and three groups were distinguished based on their sum scores: positive social influences, neutral, and negative social influences.

Perceived behaviour control was measured by one item that asked: "How sure are you that you can be regularly active?" (five-point scale, very sure – very unsure). Intention was measured with one item: "Do you plan to be regularly physical active?" (five-point scale, very likely – very unlikely).

### Socioeconomic status and other demographic characteristics

Educational attainment is only one component of the broad concept of SES, but is considered a good indicator for SES in the Netherlands [31]. Four levels of education were distinguished ((1) no education or primary education; (2) lower professional and intermediate general education; (3) intermediate professional and higher general education; (4) higher professional education and university). We also measured household income as SES-indicator, asking participants to report their net monthly household income (0–1200 euro, 1200–1800 euro, 1800–2600 euro, 2600 euro or more, and 'don't want to say/don't know'). Other demographic characteristics we measured were age (55–65, 65–75 years), sex, country of origin (Netherlands, other country), marital status (married/registered partnership, not married), and perceived general health (excellent, very good, good, moderate, poor).

### Recreational walking

Walking in leisure time was measured with the SQUASH questionnaire – a validated Dutch questionnaire to measure physical activity among an adult population [32]. Participants reported frequency (times per week), average duration (minutes per day), and intensity (low, average, high) for recreational walking over the last couple of months. However, as the distribution of the sample was highly skewed with almost one third not reporting any recreational walking (and a mean (se) of 231 (5,8) minutes recreational walking per week among those who did any recreational walking), *in*activity rather than a continuous outcome measure the focus of the current paper. The dichotomised outcome we examined was 'no recreational walking' (<10 minutes per week) vs. 'any recreational walking' (≥10 minutes per week).

### Statistical analyses

'No recreational walking' was modelled as a binary outcome variable in weighted multilevel logistic regression models of participants nested within neighbourhoods. To take into account the hierarchical nature of the data, explanatory models were run in MlwiN (version 2.02) using the logit-link function and 2nd order PQL estimation methods [33,34]. All analyses were conducted separately for education and income as SES-indicators, as they are likely to relate to different causal processes [35]. The missing value category of many explanatory factors showed high odds ratios for no recreational walking, and the prevalence of missing values was highest among participants from the lowest SES group. Therefore, to prevent overestimation of the explanatory power of these factors to SES differences in recreational walking, missing values for explanatory factors were imputed by drawing randomly from the distribution of answering categories, using observed prevalences per educational group as probabilities (analyses with non-imputed data show approximately the same results – available upon request). All bivariate and multivariate analyses were adjusted for age and sex (unless specified otherwise) and weighted (level-1 weight) to reflect our source population (i.e. older adults in the region of Eindhoven in October 2004) in terms of sex, age and educational level. This type of (single) imputation was chosen on the assumption of missing at random, dependent on SES only, i.e. Conditional Mean Imputation [36].

Firstly, we tested univariate associations of education and income with no recreational walking. Then, we examined which possible explanatory factors were significantly associated with no recreational walking (adjusted for SES, age and sex) (p < 0.05), and whether these factors were unequally distributed across SES-groups (calculated in SPSS version 11.0) [37]. Factors associated with no recreational walking and with risk categories more prevalent in low than high SES-groups were included in the following modelling sequence in MlwiN.

We examined the contribution of neighbourhood perceptions and individual cognitions to the association between SES and no recreational walking. Therefore, we firstly calculated the odds ratios of no recreational walking by socioeconomic groups adjusted for age, and sex (model 1). Then, we added neighbourhood perceptions separately (model 2); individual cognitions separately (model 3); and finally neighbourhood perceptions and individual cognitions simultaneously (model 4). When odds ratios for the SES-indicator in model 2–4 reduced (compared to model 1), this was interpreted as the contribution of the explanatory factors included in the model to socioeconomic differences in no recreational walking [38].

Also, we examined whether individual cognitions can explain the association between neighbourhood perceptions and no recreational walking. Therefore, we compared ORs for neighbourhood perceptions with and without controlling for attitude, social influences, perceived behaviour control, and intention. When the association between neighbourhood perceptions and no recreational walking attenuated after inclusion of individual cognitions in the model, we interpreted this as the explanatory role of individual cognitions to the association between neighbourhood perceptions and no recreational walking.

## Results

### Socioeconomic differences in no recreational walking

As presented in Table 1, participants in the lowest educational group (OR 1.51 (95% CI, 1.09–2.09)) and lowest income group (OR 1.33 (95% CI, 0.97–1.83)) were more likely to do no recreational walking than their higher status counterparts (unadjusted ORs). Other demographic characteristics were not associated with no recreational walking.

### Selection of explanatory factors

Three out of seven neighbourhood perceptions were significantly associated with no recreational walking, i.e. poor neighbourhood aesthetics, high social cohesion, and a small social network (see Table 2). As the latter two risk factors were more prevalent among *high *SES groups, these factors could not serve as possible explanatory factors for the raised odds for no recreational walking among *low *SES groups. All four individual cognitions were significantly associated with no recreational walking, and risk categories (i.e. negative attitude, negative social influences, low perceived behaviour control and no intention to be regularly physically active) were most prevalent among the lowest SES groups. Therefore, all individual cognitions and one neighbourhood perception (neighbourhood aesthetics) were taken into account in further explanatory models.

**Table 2:** Adjusted odds ratios (OR)^a ^for no recreational walking, and prevalence rates for response categories of neighbourhood perceptions and individual cognitions by educational level.

				Educational level				
Independent factors	OR	95% CI	p	1 (low)	2	3	4 (high)	p
NEIGHBOURHOOD perceptions								
								
*Physical neighbourhood factors*								
Neighbourhood is unsafe								
disagree	1.00		.636	95.5^b^	95.9	98.2	98.9	.004
agree	0.87	(0.49–1.55)		4.5	4.1	1.8	1.1	
Neighbourhood is unattractive								
disagree	1.00		.008	72.5	84.3	88.6	86.0	.000
agree	1.41	(1.09–1.82)		27.5	15.7	11.4	14.0	
Insufficient places for physical activity								
disagree	1.00		.256	64.3	75.2	77.5	88.7	.000
agree	1.14	(0.91–1.44)		35.7	24.8	22.5	11.3	
								
*Social neighbourhood factors*								
Social cohesion								
high	1.00		.000	38.1	36.3	41.3	40.9	.001
medium	0.62	(0.50–0.78)		30.3	37.1	38.2	34.9	
low	0.82	(0.64–1.05)		31.6	26.6	20.5	24.3	
Social network								
large	1.00		.000	34.4	37.4	37.6	29.4	.034
medium	1.56	(1.23–1.98)		33.2	32.9	40.9	37.0	
small	1.59	(1.25–2.04)		32.4	29.7	21.5	33.6	
Feeling at home in neighbourhood								
high	1.00		.120	32.4	35.3	36.4	42.3	.020
moderate	0.80	(0.64–1.01)		33.2	38.0	36.6	31.1	
low	0.99	(0.78–1.26)		34.4	26.7	27.0	26.6	
Social disorganisation								
low	1.00		.540	45.3	48.4	50.6	54.0	.000
medium	0.96	(0.75–1.22)		18.6	24.3	23.5	24.5	
high	0.86	(0.67–1.12)		36.1	27.3	25.9	21.5	
								
INDIVIDUAL cognitions								
								
Attitude towards regular physical activity								
positive	1.00		.000	25.8	32.8	36.0	32.8	.002
neutral	1.34	(1.07–1.67)		61.9	54.9	58.5	58.4	
negative	4.16	(2.96–5.84)		12.3	12.3	5.4	8.7	
Social influences for regular physical activity								
positive	1.00		.000	54.9	50.1	49.6	53.2	.003
neutral	1.62	(1.32–1.99)		30.3	40.3	43.9	38.3	
negative	1.76	(1.26–2.45)		14.8	9.6	6.5	8.5	
Perceived behaviour control to be regularly active								
(very) sure	1.00		.000	59.2	67.0	70.7	74.2	.001
not sure/unsure	1.65	(1.32–2.07)		31.0	25.7	20.5	19.0	
(very) unsure	2.10	(1.48–2.97)		9.8	7.3	8.8	6.8	
Intention to be regularly active								
yes	1.00		.000	45.9	55.0	57.3	67.0	.000
maybe	1.84	(1.49–2.27)		38.9	38.1	35.2	26.7	
no	4.41	(3.09–6.29)		15.2	6.9	7.5	6.4	

^a ^Models were weighted, and adjusted for age, sex, and educational level.

^b ^This is the percentage of respondents in a certain response category per socioeconomic group; for example, 95.5% of those in the lowest group disagreed with the statement "My neighbourhood is unsafe".

### Explaining the 'SES – no recreational walking' association

As presented in Table 3, the sex- and age-adjusted OR to do no recreational walking for the lowest educational group (OR 1.67 (95% CI, 1.18–2.35) attenuated when neighbourhood aesthetics was included in the model (model 2), or when individual cognitions were included (model 3), and further reduced when all these factors together (model 4) were taken into account (OR 1.30 (95% CI, 0.91–1.87). Attitude and intention regarding regular physical activity had the largest effect on the reduction of SES inequalities in recreational walking. The odds to do no recreational walking were lowest for the second-highest educational group in all models.

**Table 3:** Odds ratios with 95% confidence intervals (OR, 95% CI) for no recreational walking by education, mediated by neighbourhood perceptions and individual cognitions.

		Model 1 (base model): education + age + sex	Model 2: base + neighbourhood	Model 3: base + individual	Model 4: base + neighbourhood + individual
*Education*	*%* *no walking*	OR (95% CI)	OR (95% CI)	OR (95% CI)	OR (95% CI)
1 – low (n = 281)	38.5	1.67 (1.18–2.35)	1.60 (1.13–2.27)	1.33 (0.93–1.90)	1.30 (0.91–1.87)
2 – (n = 908)	34.7	1.49 (1.17–1.90)	1.48 (1.16–1.89)	1.35 (1.04–1.75)	1.29 (0.99–1.68)
3 – (n = 366)	22.1	0.84 (0.60–1.18)	0.84 (0.60–1.19)	0.80 (0.57–1.13)	0.75 (0.53–1.06)
4 – high (n = 439)	29.2	1.00	1.00	1.00	1.00
					
*Neighbourhood perceptions*					
					
My neighbourhood is unattractive					
disagree			1.00		1.00
agree			1.32 (1.06–1.65)		1.19 (0.95–1.50)
					
*Individual cognitions*					
					
Attitude towards regular physical activity					
positive				1.00	1.00
neutral				1.12 (0.87–1.45)	1.11 (0.86–1.43)
negative				2.30 (1.59–3.32)	2.26 (1.57–3.26)
					
Social influences for regular physical activity					
positive				1.00	1.00
neutral				1.24 (1.01–1.52)	1.24 (1.02–1.532)
negative				1.54 (1.11–2.14)	1.54 (1.11–2.14)
					
Perceived behaviour control to be regularly active					
(very) sure				1.00	1.00
not sure/unsure				1.21 (0.95–1.55)	1.20 (0.94–1.54)
(very) unsure				1.57 (1.11–2.22)	1.56 (1.10–2.21)
					
Intention to be regularly active					
(very) likely				1.00	1.00
maybe				1.31 (0.99–1.73)	1.30 (0.98–1.72)
(very) unlikely				2.38 (1.59–3.57)	2.38 (1.59–3.57)
					
Random effects ^a^					
Level-2 variance (SE)		0.000 (0.000)	0.000 (0.000)	0.000 (0.000)	0.000 (0.000)

^a ^Weighted multilevel models were estimated with the iterative generalized least squares procedure implemented in MlwiN version 2.02.

Results of the analyses with income as SES-indicator showed the same pattern as those for education. However, there was a smaller socioeconomic gradient for income (see model 1, Table 4), and the socioeconomic differences were fully explained when all explanatory factors were taken into account (model 4, Table 4). People who ticked the answer category "I do not want to report my income, or I do not know" were most likely not to engage in any recreational walking.

**Table 4:** Odds ratios with 95% confidence intervals (OR, 95% CI) for doing no recreational walking by income, adjusted for neighbourhood perceptions and individual cognitions.

		Model 1 (base model): income + age + sex	Model 2: base + neighbourhood	Model 3: base + individual	Model 4: base + neighbourhood + individual
*Income*	*% no walking*	OR (95% CI)	OR (95% CI)	OR (95% CI)	OR (95% CI)
1 – low (n = 303)	35.7	1.40 (0.98–2.01)	1.34 (0.94–1.91)	1.03 (0.73–1.47)	1.01 (0.71–1.42)
2 – (n = 535)	30.3	1.10 (0.81–1.49)	1.09 (0.80–1.48)	0.94 (0.70–1.27)	0.93 (0.69–1.26)
3 – (n = 515)	27.8	0.81 (0.60–1.10)	0.81 (0.60–1.10)	0.77 (0.57–1.05)	0.77 (0.57–1.05)
4 – high (n = 429)	29.4	1.00	1.00	1.00	1.00
5 – don't want to say/don't know (n= 243)	36.6	1.32 (0.97–1.81)	1.31 (1.05–1.63)	1.16 (0.85–1.58)	1.15 (0.84–1.56)
					
*Neighbourhood perceptions*					
					
My neighbourhood is unattractive					
disagree			1.00		1.00
agree			1.31 (1.05–1.63)		1.19 (0.95–1.50)
					
*Individual cognitions*					
					
Attitude towards regular physical activity					
positive				1.00	1.00
neutral				1.11 (0.86–1.43)	1.10 (0.85–1.42)
negative				2.32 (1.61–3.34)	2.28 (1.59–3.28)
					
Social influences for regular physical activity					
positive				1.00	1.00
neutral				1.22 (0.99–1.51)	1.23 (1.001.51)
negative				1.53 (1.10–2.11)	1.53 (1.10–2.12)
					
Perceived behaviour control to be regularly active					
(very) sure				1.00	1.00
not sure/unsure				1.25 (0.98–1.59)	1.24 (0.97–1.57)
(very) unsure				1.55 (1.09–2.20)	1.55 (1.09–2.19)
					
Intention to be regularly active					
(very) likely				1.00	1.00
maybe				1.32 (1.00–1.75)	1.31 (0.99–1.74)
(very) unlikely				2.43 (1.65–3.57)	2.43 (1.65–3.57)
					
Random effects ^a^					
Level-2 variance (SE)		0.000 (0.000)	0.000 (0.000)	0.000 (0.000)	0.000 (0.000)

^a ^Weighted multilevel models were estimated with the iterative generalized least squares procedure implemented in MlwiN version 2.02.

### Explaining the 'neighbourhood aesthetics- no recreational walking' association

The association between neighbourhood aesthetics and no recreational walking reduced to non-significance when individual cognitions were taken into account (model 4, Table 3 and Table 4), although the OR for no recreational walking among those finding their neighbourhood unattractive remained elevated (OR 1.19 (95% CI, 0.95–1.50).

## Discussion

This study is among the first to investigate how neighbourhood perceptions and individual cognitions contribute to socioeconomic differences in recreational walking among older adults using a multilevel design. We found the lowest socioeconomic group most likely to be inactive regarding recreational walking, which is consistent with previous studies on walking [9,10] and other physical activity outcomes [39-43]. Also consistent with other findings, we found that neighbourhood perceptions (i.e. neighbourhood aesthetics [9,10,43]) and individual cognitions (i.e. attitude, social influences, perceived behaviour control, and intention [9,43]) were important in the explanation of socioeconomic differences in recreational walking. Associations of neighbourhood factors with recreational walking, and their contribution to socioeconomic differences in recreational walking were smaller than the effect and contribution of individual factors (similar to findings for sports participation [44]). Still, as small odds ratios for neighbourhood characteristics may imply that changes to (perceptions of) the neighbourhood context may have a significant effect on physical activity levels, these may offer important opportunities to reduce socioeconomic differences in physical activity.

Going beyond previous studies, our findings suggested that perceived unfavourable neighbourhood aesthetics contributed to the SES-inactivity relationship *via *individual physical activity cognitions (since the OR for neighbourhood aesthetics reduced to non-significance when individual cognitions were taken into account, OR= 1.19 (95% CI: 0.95–1.50)). As the OR for neighbourhood aesthetics remained somewhat elevated, a direct effect of neighbourhood aesthetics may also play a role. These results support the hypothesis of ecological models of physical activity [12,13], which suggest that environmental factors show both direct and indirect effects with physical activity. Findings indicated that older adults from socioeconomically disadvantaged backgrounds were more likely to perceive poor neighbourhood aesthetics, which in turn may have reduced their perceived behavioural control expectations and may have had a negative impact on their attitudes toward regular physical activity, explaining their lower levels of recreational walking. Previous studies also reported (small) mediating effects of attitude and perceived behaviour control**/**self-efficacy in the association between environmental influences and physical activity [45-47].

The main strength of our study is that we could estimate the contribution of a wide range of physical and social neighbourhood perceptions and individual cognitions to the explanation of socioeconomic differences in recreational walking among older adults, using multilevel analysis techniques to correct for possible area effects. However, there were several limitations of our study. First, the cross-sectional design precluded any causal inferences from being drawn. Mediation effects only indicated that causal pathways may exist, however, selection may also play a role, i.e. people that find regular physical activity important may choose to live in a pleasant environment. Also, as neighbourhood attractiveness and individual cognitions were both self-reported, other characteristics (e.g. personality, depressiveness) may have influenced both factors in the same (positive/negative) way. Due to the exclusion of participants with missing values for recreational walking, education, and household income, this study may have underestimated SES-walking associations, as lower SES groups may have been more inclined towards selective non-response. Furthermore, we could not examine objective, level-2 measures of neighbourhood influences, and therefore, it remains uncertain to what extent SES differences in neighbourhood perceptions reflect objective differences between neighbourhoods. However, in additional multilevel analyses we found significant clustering of perceived safety, attractiveness and availability of facilities within neighbourhoods, even when adjusting for resident's age, sex, and education. This clustering of perceptions might indicate true neighbourhood differences (results available on request). Individual cognitions were not measured behaviour-specific for recreational walking, but referred to regular physical activity ("being physically active with moderate intensity for at least 30 minutes per day"). In addition, neighbourhood perceptions were not specifically asked in the context of recreational walking. Increased specificity in and correspondence between outcome, and individual and neighbourhood variables, may lead to stronger associations and increased explanation of socioeconomic differences in recreational walking [15].

Simple cross tabulations indicated that the proportion of residents engaging in recreational walking does significantly vary by neighbourhood (results available upon request). Unexpectedly, we did not find any neighbourhood variance in recreational walking within our multilevel models (see Table 3 en Table 4), which is difficult to explain. The multilevel statistical package MlwiN (version 2.02) was used nonetheless, as explanatory factors did cluster within neighbourhood.

We found opposite associations of social cohesion and social network with recreational walking: both *high *social cohesion and a *small *social neighbourhood network were associated with a lower likelihood of recreational walking. The latter association was expected and in line with the literature [28,40]: participants with a small social neighbourhood network may find it more difficult to find company for recreational walking, or may experience less social support/peer encouragement for physical activity. However, one can only speculate why people who experience high social cohesion (i.e. those who reported that people in the neighbourhood are willing to help each other, and that people in the neighbourhood agree on norms and values) are more likely to do no recreational walking. Maybe neighbourhoods with high social cohesion organized other neighbourhood activities in which these participants engaged rather than walking. Or, if social cohesion is high but the social norm is *not *to engage in recreational walking, people may find it more difficult to go walking than those living in neighbourhoods with low social cohesion and no norm regarding walking.

## Conclusion

This study is among the first to show that unfavourable neighbourhood perceptions contribute to the explanation of socioeconomic differences in no recreational walking among older adults mainly indirectly, i.e. via unfavourable individual cognitions towards regular physical activity. More research into causal pathways between (objective and perceived) neighbourhood influences and individual cognitions is needed to better understand how socioeconomic disadvantage leads to physical inactivity. Our results suggest that intervention and policy strategies to reduce socioeconomic differences in lack of recreational walking among older adults would need to intervene on both neighbourhood perceptions as well as individual cognitions.

## Competing interests

The authors declare that they have no competing interests.

## Authors' contributions

CBMK conceived of the specific study as described in this paper, coordinated data collection, performed the statistical analyses, and drafted the manuscript. FJ designed the GLOBE study, coordinated data collection, had critical input in the data analyses, and helped drafting the manuscript. KG coordinated data collection, had critical input in the data analyses, and helped drafting the manuscript. MH helped drafting the manuscript. JB had critical input in coordination of the data collection, and drafting the manuscript. JPM designed the GLOBE study, and had critical input in coordination of the data collection, analyses and drafting the manuscript. All authors read and approved the final manuscript.
